# Supplementary material for: The Bone Regeneration Capacity of BMP-2 + MMP-10 Loaded Scaffolds Depends on the Tissue Status
Source: Pharmaceutics. 2021 Jun 29;13(7):979. doi: 10.3390/pharmaceutics13070979 (PMC8308972; doi:10.3390/pharmaceutics13070979)
Supplement: Supplementary file 1 [file pharmaceutics-13-00979-s001.zip › pharmaceutics-1264501-supplementary.pdf]

pharmaceutics

Supplementary Material: The Bone Regeneration Capacity of BMP-2 + MMP-10 Loaded Scaffolds Depends on the Tissue Status

Patricia Garcia-Garcia, Ricardo Reyes, José Antonio Rodriguez, Tomas Martín , Carmen Evora , Patricia Díaz-Rodríguez and Araceli Delgado

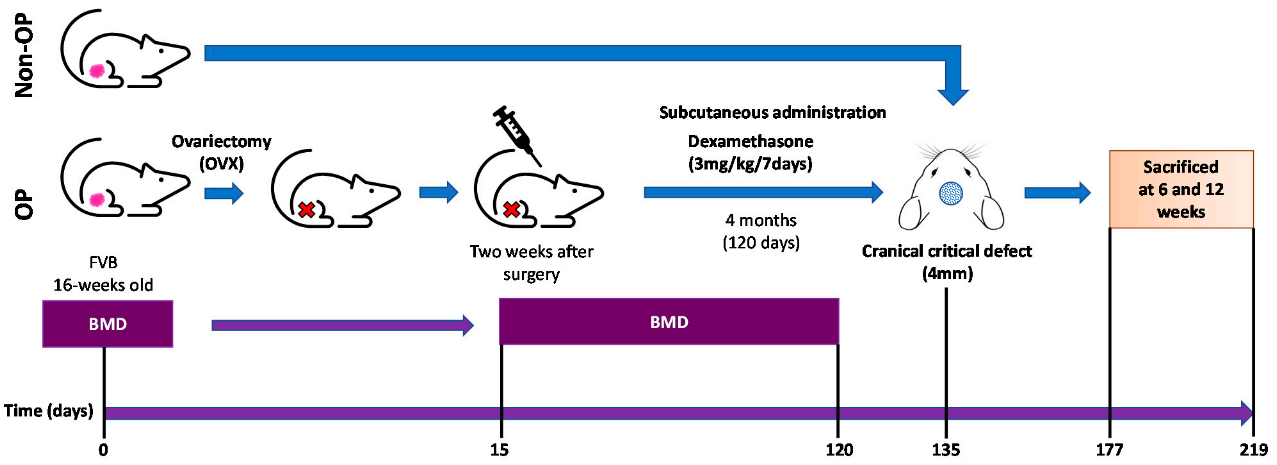

Figure S1. - *In vivo* experiments timeline for osteoporotic model evaluation by bone mineral density (BMD) measurements and the assessment of scaffold performance.

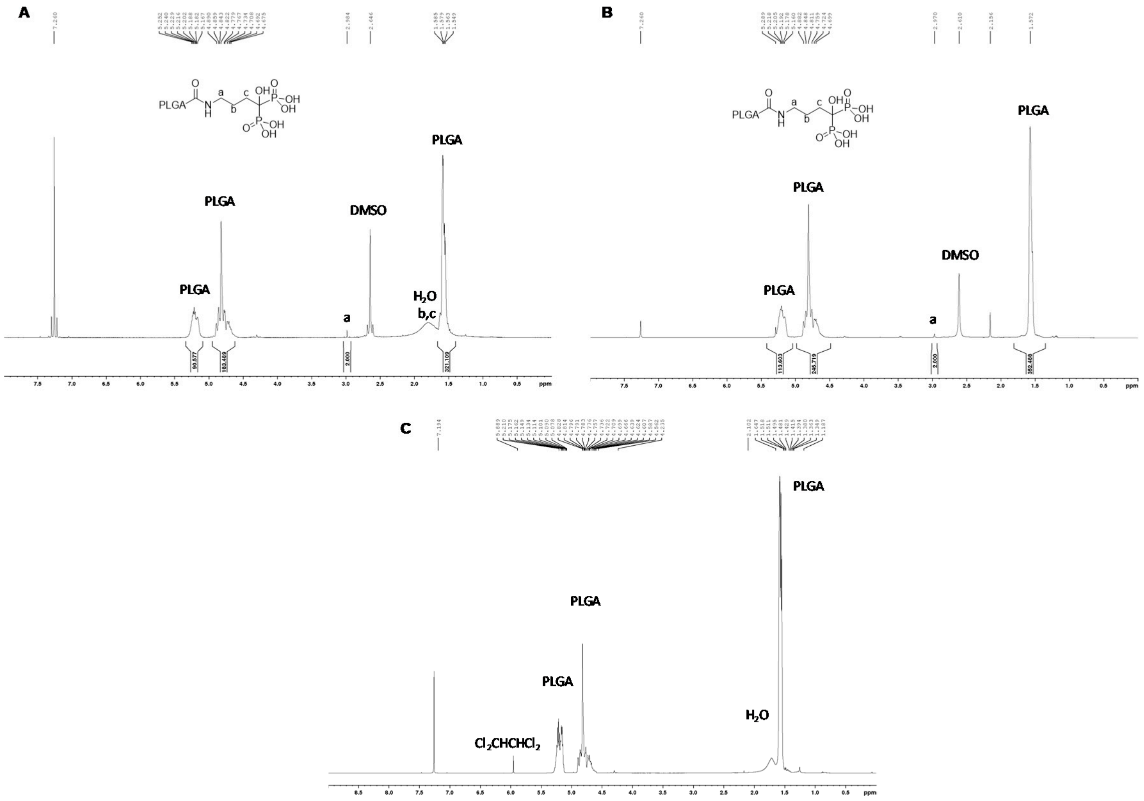

Figure S2. - ^{1}H-NMR spectrum of **A**) PLGA-ALD from Resomer® RG 502 H; **B**) PLGA-ALD from Resomer® RG 504 H and **C**) Electrospun meshes soaked 72 hours in water ($Cl_2CHCHCl_2$ was used as internal standard). The signal at 7.26 ppm corresponds to the signal from the solvent $CHCl_3$.

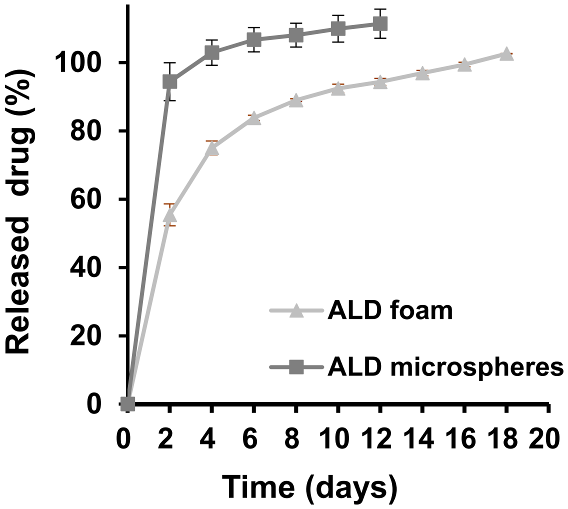

Figure S3. - In vitro drug release profiles of ALD from microsphere-loaded chitosan foams (ALD foam) and plain microspheres (ALD microspheres).
